# Does hydroxychloroquine reduce the risk of infection in patients with systemic lupus erythematosus? a systematic review and meta-analysis

**DOI:** 10.1371/journal.pone.0320353

**Published:** 2025-03-25

**Authors:** Shangtian Wang, Maojia Ka, Wenying Wu

**Affiliations:** Department of Rheumatology and Immunology, The First People’s Hospital of Xining, Xining, China; Universita Campus Bio-Medico di Roma, ITALY

## Abstract

**Objectives:**

This study aimed to investigate the anti-infective utility of hydroxychloroquine in patients with systemic lupus erythematosus (SLE) by analyzing published case-control and cohort studies.

**Methods:**

A systematic literature review was conducted on January 28, 2024, using PubMed, Cochrane, Embase, and Web of Science Core Collection databases. Odds ratios (OR) were used for statistical analysis.

**Results:**

Hydroxychloroquine exhibits a propensity to diminish infection risk in systemic lupus erythematosus patients, albeit without statistical significance (OR = 0.77, 95%CI 0.51-1.18, p = 0.23). Subgroup analyses revealed a significant prevention of serious infections (OR = 0.40, 95%CI 0.25-0.64, p = 0.0001). Interestingly, a potential causal relationship between hydroxychloroquine use and lower infection risk was observed in the cohort studies subgroup (OR = 0.66, 95%CI 0.44-0.99, p = 0.04), but not in the case-control studies subgroup (OR = 1.06, 95%CI 0.63-1.79, p = 0.83). It is important to note the risks associated with high-dose use, such as retinopathy.

**Conclusions:**

Although hydroxychloroquine tends to reduce infection risk in SLE patients, the evidence is not strong. It can decrease severe infections, but high doses should be used cautiously and selectively in patients with impaired renal function. Further studies are required to establish optimal dosing and efficacy for specific diseases, considering the potential influence of study design on the observed associations between hydroxychloroquine use and infection risk in SLE patients.

## Introduction

Systemic lupus erythematosus (SLE), a chronic autoimmune disease, affects multiple systems. Inflammatory organ disease, caused by autoantibody production and immune complex deposition in tissues, characterizes SLE. SLE carries serious cardiovascular and renal risks due to the variable morphology of the affected organs [1]. Early studies showed a 5-year survival rate of only 50% [2] and a 10-year survival rate of 63.2% [3]. Glucocorticoids, cyclophosphamide, azathioprine, and cyclosporine are typically used in clinical treatments. These drugs, used for over 30 years, pioneered clinical therapeutic treatments. Since 2000, in-depth research on disease mechanisms has led to the introduction and clinical application of various biologics, such as rituximab, belemucirumab, and amfilimumab. These biologics have significantly increased patient survival rates. Consequently, due to this favorable advancement, the survival rate surpassed 90% in 2010 [4]. Despite improved efficacy, infections still account for 31.7% to 36.5% of deaths in SLE patients [4,5]. Thus, infection prevention is crucial for prognosis and treatment. A multipurpose drug with good therapeutic efficacy, low side effects, anti-infection properties, and affordability would be highly advantageous in this situation. Hydroxychloroquine appears to fulfill these criteria.

Hydroxychloroquine, a quinacrine derivative, was first introduced as an antimalarial drug in 1955 [6]. Its safety and long-term beneficial effects have led to its widespread use in treating autoimmune diseases, such as rheumatoid arthritis and systemic lupus erythematosus (SLE) [7–11]. Based on accumulated application practices rather than subjective opinions, hydroxychloroquine is considered a therapeutic agent worth trying for all SLE patients. As all antimalarial drugs possess broad antimicrobial properties [12], it can be inferred that hydroxychloroquine may effectively control infectious diseases in SLE patients. While some studies support this inference [13–16], a definitive conclusion has not been reached. The recent emergence of new coronaviruses has rekindled interest in the relationship between hydroxychloroquine and infection risk in SLE patients. This study aims to summarize published cohort and case-control studies, investigating the relationship between hydroxychloroquine and infection risk in SLE patients, and to explore differences based on infection type, analysis type, study type, and dose used.

## Materials and methods

### Literature search

This study followed the PRISMA (Preferred Reporting Items for Systematic Reviews and Meta-Analyses) checklist published in 2020 [17] (S1 Table) and was registered in the PROSPERO system (CRD42024526670). A systematic literature search was conducted in four databases (PubMed, Cochrane, Embase, and Web of Science Core Collection) with a cut-off date of January 28, 2024. The search was performed in English using the keywords “hydroxychloroquine,” “infection,” and “systemic lupus erythematosus” (S2 Table). At least two rheumatology clinicians conducted multiple rounds of manual review on all relevant literature to ensure relevance, resolve disagreements, and meet the inclusion criteria.

### Inclusion and exclusion criteria

The analyses included only formally published case-control or cohort studies that met the following criteria: (1) the patients were adults with SLE and hydroxychloroquine was used as an intervention; (2) the studies were published in English; and (3) the results of the studies were expressed in terms of Odds ratio (OR), Relative risk (RR), Hazard ratio (HR), and their respective 95% confidence intervals (95% CIs), or there was sufficient information to enable us to calculate the results. All the raw data depicted in Supplementary File 1.

### Data extraction

Two independent researchers (S.W and M.K) performed the data extraction process. Disagreements arising during the process were resolved through discussion. Extracted data from the included studies encompassed first author, publication year, study country, design, sample size, gender, age, adjustment variables, hydroxychloroquine dose, outcomes, and OR/RR/HR (95% CIs)

### Quality assessment

The included case-control and cohort studies were independently assessed using the Newcastle-Ottawa Scale (NOS). Studies scoring 7-9 were considered high quality [18]. As with data extraction, disagreements were resolved through discussion.

### Statistical analysis

Meta-analyses were performed using Review Manager version 5.4.1 (Cochrane Collaboration, Oxford, UK), uniformly assessing the Odds ratio (OR). For all outcome metrics, 95% confidence intervals (95% CI) were calculated, and heterogeneity between studies was estimated using the inconsistency index (I^2^) [19]. Significant heterogeneity was considered when *p* <  0.05 or I^2^ >  50%. All data were analyzed using a random effects model. Funnel plots were created using Review Manager 5.4.1 (Cochrane Collaboration, Oxford, UK) when data from ≥ 10 studies were available. Potential publication bias was evaluated using Egger’s regression tests with Stata version 15.0 (Stata Corp, College Station, Texas, USA). Statistical significance was set at *p* <  0.05. Multivariate data were used for all analyses, except for univariate data in the analysis type subgroups.

## Results

### Study characteristics and results of the screening process

**Fig 1:**
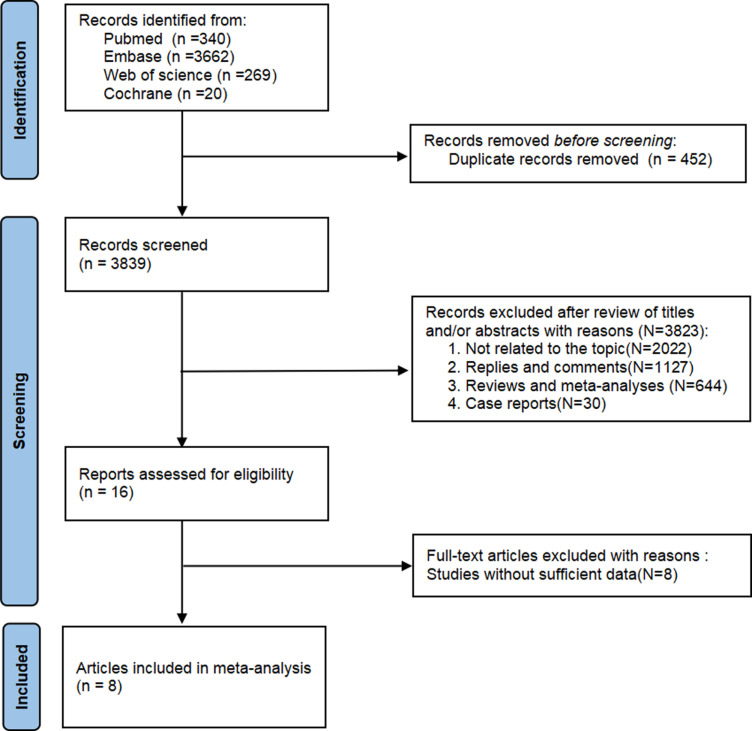
Flowchart of literature screening process.

The literature screening methodology and process are displayed in Fig 1. The search yielded a total of 4,291 publications: 340 from PubMed, 3,662 from Embase, 20 from Cochrane, and 269 from Web of Science Core Collection. A total of 452 duplicates were deleted, some of which were repeated more than twice, and literature was deleted according to the type of literature, including 714 conference abstract articles, 28 conference paper articles, 1 conference review article and 33 Editorial articles. There were 289 letters, 61 notes, 1 preprint, 644 reviews and 30 short surveys. 2022 literatures were deleted based on title and abstract, and 8 literatures could not extract data. Finally, eight studies [20–27] were included for analysis after excluding publications that did not meet the inclusion and exclusion criteria(Supplementary File 2). The analysis included five cohort studies [20–24] and three case-control studies [25–27]. The studies investigated four infectious factors (severe infections, hospitalised infections, herpes zoster, and COVID-19 infections) in populations from three continents (North America, Asia, and Europe). The basic characteristics of each study are presented in Table 1.

**Table 1:** Basic characteristics of included literature.

Study	Year of publication	Country	Study design	Group	Sample of Cases (Experiment)	Sample of Controls	Age of Cases(years)	Age of Controls (years)	Gender of Cases(F/M)	Gender of Control(F/M)	Duration of study	Variables of adjustment	Hydroxychloroquine (mg/day,mean±sd,number(%))	OR, RR or HR(95% CI)	Outcomes
**Merayo-Chalico**	2013	Mexico	case-control	Case: SLE with severe infection; Control: SLE without severe infection	89	78	33.3 ± 11	34.4 ± 11.5	71/18	71/7	6 years	Lymphopenia; Neutropenia; Time since diagnosis; Anti-dsDNA; C3; C4; SLEDAI score; PDN treatment;CFM treatment;AZA treatment;MMF treatment	65.1 ± 11.8/91 ± 11.5	0.542(0.25-1.1)	severe infections
**Hu**	2016	Taiwan	case-control	Case: SLE with HZ; Control: SLE without HZ	1555	3049	36.44 ± 16.87	36.64 ± 16.7	1382/173	2730/319	14 years	age, sex, income category, urbanization level, diabetes mellitus, lymphoma, leukemia, breast cancer, liver cancer, HIV infection, liver cirrhosis, renal disease, cerebrovascular disease, myocardial infarction, and use of other immunosuppressive medications	≤216	1.41(1.17-1.71)	Herpes zoster
**Hu**	2016	Taiwan	case-control	Case: SLE with HZ; Control: SLE without HZ	1555	3049	36.44 ± 16.87	36.64 ± 16.7	1382/173	2730/319	14 years	age, sex, income category, urbanization level, diabetes mellitus, lymphoma, leukemia, breast cancer, liver cancer, HIV infection, liver cirrhosis, renal disease, cerebrovascular disease, myocardial infarction, and use of other immunosuppressive medications	>216	2.06(1.74-2.44)	Herpes zoster
**Sakia**	2020	Japan	cohort	Case: SLE with HCQ treatment; control:SLE without HCQ treatment	–	–	–	–	–	–	8 months	Age by decade; Female; Diabetes mellitus; Heart failure; Chronic kidney disease; Pulmonary disease; Steroid pulse therapy; Oral CS dosage(mg/day); MMF use	200 ± 74.074	0.87(0.57,1.31)	Hospitalized infection
**Zamora**	2020	Philippines	case-control	Case: SLE with HZ; control: SLE without HZ	65	130	36.75 ± 1.35	36.75 ± 0.95	61/4	122/8	6 years	IV cyclophosphamide; Mycophenolate mofetil; Methylprednisolone; pulse therapy; Prednisone; Renal involvement; SELENA‐SLEDAI > 3	156.92 ± 15.50	0.13(0.03-0.49)	Herpes zoster
**Simard**	2021	Sweden	cohort	SLE with HCQ; SLE with DMARD	–	–	–	–	–	–	6.2 years	nephritis, corticosteroids, history of infusion, history of infection	–	0.77(0.51,1.16)	Hospitalized infection
**Hidekawa**	2023	China	cohort	SLE with HCQ; SLE without HCQ		–	–	–	–	–	5 years	–	–	0.590(0.329,1.058)	severe infections
**Patil**	2023	India	cohort	SLE with COVID; SLE no COVID	–	–	–	–	–	–	10months	–	–	1.20(0.47-3.06)	COVID
**Sun**	2023	Taiwan	cohort	SLE with HCQ; SLE with other medicines	–	–	–	–	–	–	10 year and 6 months	SLEDAI-2K > 12; Hemoglobin < 8g/dL; Platelet < 150 × 109/L; Platelet < 150 × 109/L; sERUM IgG < 7.51g/L; Chronic kidney disease; Prednisolone eauivalent ≧ 15mg/day; Azathioprine; Mycophenolate;Calcineurin inhibitors	–	0.35(0.15,0.82)	severe infections

### Assessment of study quality

Table 2 displays the quality scores of the included studies. Three studies received a score of 9 [20,21,26]. Of the remaining seven studies, three received a score of 8 [23,26,27] due to confounding factors, while the remaining two received a score of 7 [22,24] due to confounding factors and shorter follow-up time. (Table 2) And the details of quality assessment was depicted in Supplementary File 3.

**Table 2:** Literature Quality Assessment Results of Included Studies.

Study	Study design	Selection	Comparability	Outcome Scores	NOS total Score
**Simard 2021**	Cohort	4	2	3	9
**Hu 2016**	Case-control	4	2	3	9
**Sakai 2020**	cohort	4	2	3	9
**Zamora 2020**	Case-control	4	1	3	8
**Merayo-Chalico 2013**	Case-control	4	1	3	8
**Patil 2023**	Cohort	4	1	3	8
**Hidekawa 2023**	Cohort	3	2	2	7
**Sun 2023**	Cohort	4	2	1	7

### Outcomes of meta-analysis

#### Results of overall analysis.

**Fig 2:**
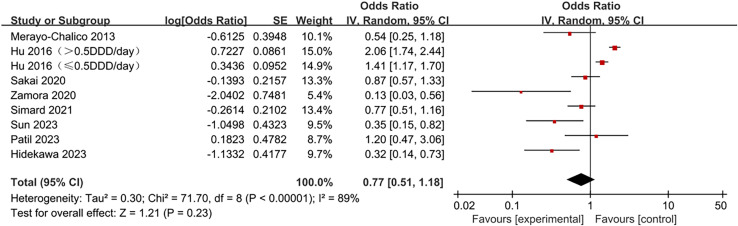
Forest plot of overall analysis results. Note: DDD: defined daily dose.

The pooled risk estimates of hydroxychloroquine on infection risk in SLE patients are displayed in Fig 2. Hydroxychloroquine did not significantly reduce infection risk in SLE patients (OR =  0.77, 95% CI 0.51-1.18, *p* =  0.23), with observed heterogeneity (I^2^ =  89%, *p* <  0.00001).

#### Results of subgroup analyses.

**Fig 3:**
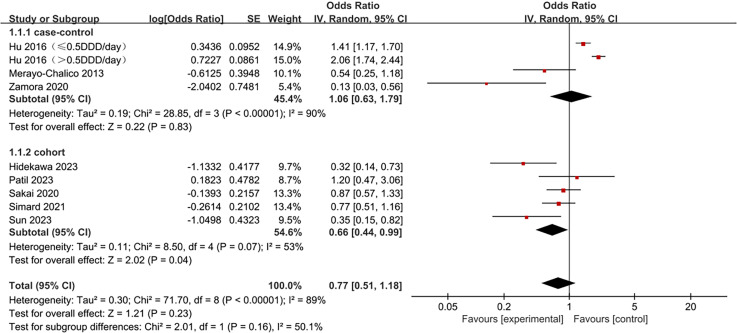
Forest plot for subgroups of study types. Subgroup analyses were conducted on five studies, including four univariate and five multivariate analyses. Hydroxychloroquine significantly reduced infection risk in SLE patients in the univariate subgroup (OR =  0.32, 95% CI 0.22-0.47, *p* <  0.00001) but not in the multivariate subgroup (OR =  0.69, 95% CI 0.40-1.20, *p* =  0.19). The multivariate subgroup displayed significant heterogeneity (I^2^ =  91%, *p* <  0.00001), while the univariate subgroup showed no heterogeneity (I^2^ =  0%, p =  0.53) (Fig 4).

The study type subgroup included eight studies: five cohort studies and three case-control studies. The overall analysis results corroborated significant effectiveness (OR =  0.48, 95% CI 0.28-0.82, p =  0.007, I^2^ =  92%, *p* <  0.00001), yet subgroup variations were observed. In the cohort study subgroup, hydroxychloroquine significantly reduced infection risk in SLE patients (OR =  0.66, 95% CI 0.44-0.99, *p* =  0.04), but not in the case-control study subgroup (OR =  1.06, 95% CI 0.63-1.79, *p* =  0.83). Heterogeneity is present but not statistically significant in cohort study subgroups (I^2^ =  53%, *p* =  0.07) but was significant in the case-control study subgroup (I^2^ =  90%, p <  0.00001) (Fig 3).

**Fig 4:**
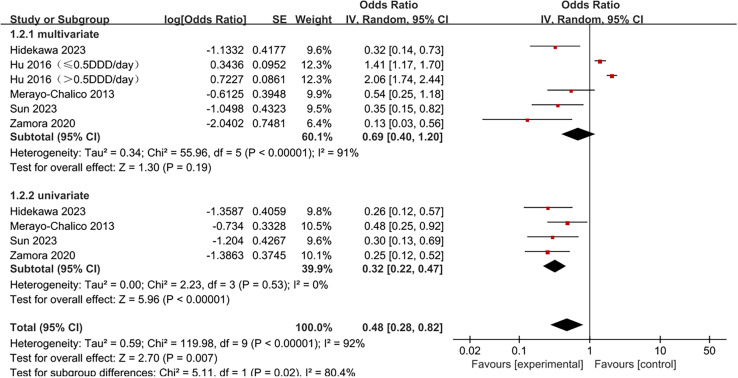
Forest plot for subgroups of analysis types. Eight studies were divided into four subgroups based on infection type for subgroup analysis: severe infections, hospitalized infections, herpes zoster, and COVID-19 infections. As shown in Fig 5, hydroxychloroquine significantly reduced infection risk in SLE patients in the severe infection subgroup (OR =  0.40, 95% CI 0.25-0.64, *p* =  0.0001). However, this conclusion could not be drawn for the other subgroups. Significant heterogeneity was observed only in the herpes zoster infection subgroup (I^2^ =  90%, *p* <  0.0001) and could not be assessed in the COVID-19 infection subgroup. No significant heterogeneity was observed between the severe infection and hospitalized infection subgroups.

**Fig 5:**
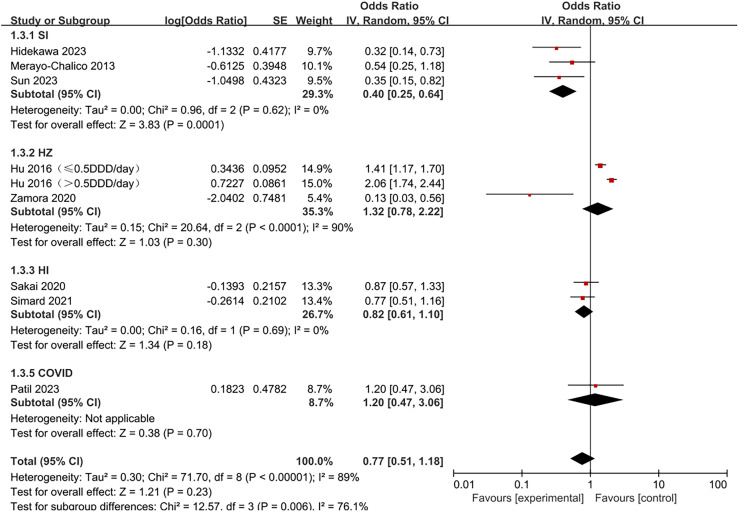
Forest plot for subgroups of infection types.

### Sensitivity analysis

**Fig 6:**
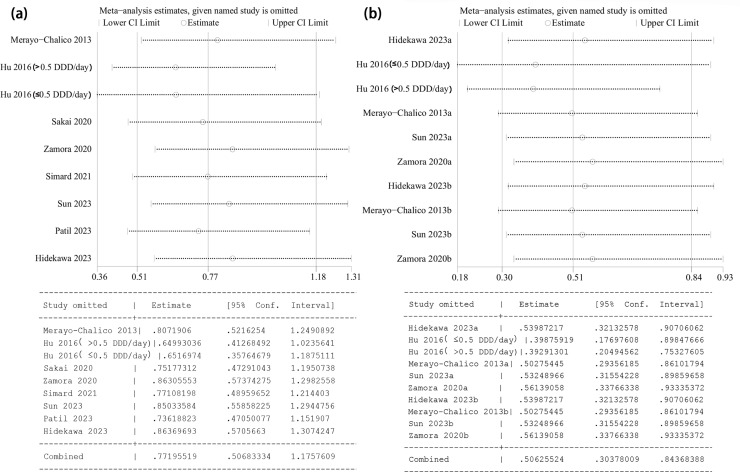
Sensitivity analysis results. (a) Overall analyses and subgroup analyses (except for subgroups of analysis types), (b) Subgroups of analysis types. Note: Data ending in “a” or unmarked are multivariate, those ending in “b” are univariate”.

One-way sensitivity analyses were performed on the results. The study type, infection type subgroups were represented using the same graph due to their consistency with the data used for the overall analysis. The results remained stable and unaffected regardless of the excluded studies (Fig 6).

## Discussion

SLE patients have defects in their immune systems, and mainstream immunosuppressive therapies increase their infection risk. In this context, a multi-effective drug with excellent efficacy, mild side effects, significant anti-infective capacity, and affordability would play a crucial role. Hydroxychloroquine, as one such candidate, appears to meet these criteria perfectly.We conducted a meta-analysis of eight studies (three case-control and five cohort) to evaluate the effectiveness of hydroxychloroquine in preventing infections, considering its common use in SLE patients and wide antimicrobial efficacy. The analysis indicated that hydroxychloroquine tended to reduce infection risk in SLE patients, although not statistically significant. Hydroxychloroquine may pose a risk with increasing doses. Subgroup analyses revealed that hydroxychloroquine played an important role in responding to serious infections. The study type subgroup showed that hydroxychloroquine use might be associated with a reduced infection risk in SLE patients. The analysis type subgroup showed that hydroxychloroquine significantly reduced infection risk in SLE patients when used univariately.

Pulmonary and urinary tract infections are the most common types of serious infections, as shown by numerous studies. Escherichia coli, Staphylococcus aureus, Mycobacterium, and Cryptococcus are the most common causative bacterial species in serious infections [20,22,24,27]. Hydroxychloroquine effectively inhibits their survival through pH-dependent iron deprivation and direct toxicity by increasing phagolysosomal pH [12,28,29]. Enterococcus spp, Streptococcus pneumoniae, Pseudomonas aeruginosa, Klebsiella pneumoniae, and other common nosocomial infection-causing organisms have also been found in patients with severe infections. Unfortunately, hydroxychloroquine has demonstrated unsatisfactory efficacy in targeting these pathogenic infections, as evidenced by multiple real-life cases [30–33]. The efficacy of hydroxychloroquine in treating herpes zoster and COVID-19 is not significant and remains controversial. Clinical use of hydroxychloroquine against these pathogens requires careful consideration. Most of the aforementioned pathogens are multidrug-resistant (MDR) and may require additional therapeutic agents to achieve better outcomes. A recent antimicrobial trial involving non-antibiotic drugs, specifically metformin, haloperidol, and hydroxychloroquine, in combination with antibiotics has shown enhanced antimicrobial activity. This could provide valuable clinical tools for addressing this complex condition [34].

Monotherapy is not practical for SLE patients. SLE, a disease with multisite involvement, is inevitably treated with co-administration of glucocorticosteroids, immunosuppressive agents, and other clinical therapeutic agents, despite hydroxychloroquine demonstrating significant utility in a unifactorial subgroup. Patients with infections tend to use glucocorticosteroids (e.g., prednisone) and immunosuppressants (e.g., cyclophosphamide) more frequently than antimalarials (e.g., hydroxychloroquine), as demonstrated by research. Even when patients opt for antimalarials, the dosage is often relatively low, increasing infection risk and interfering with hydroxychloroquine’s effectiveness in preventing infection [35,36]. Therefore, we recommend careful consideration of the drugs to be used alongside hydroxychloroquine.

The balance between dosage and efficacy of hydroxychloroquine, a highly debated topic, should be considered in light of its potential side effects. For further discussion, we ranked the five studies reporting dosage data from highest to lowest. We found that neither the highest dose (273 ±  74.074 mg/day) nor the lowest dose (65.1 ±  11.8 mg/day) showed significant utility. However, the three studies with intermediate dosages showed significance, though significance does not equate to favorable results. The two groups with relatively high dosage (216 mg/day) and relatively low dosage (158.82 ±  15.5 mg/day) exhibited opposite trends. Thus, we agree with Zamora et al. [25] that higher doses of hydroxychloroquine may achieve significant efficacy through unknown mechanisms. However, we also concur that there is an upper limit to high doses, and exceeding it may be counterproductive.At least four societies recognize that the low-risk dose of hydroxychloroquine for treating SLE is no more than 5 mg/kg/day (calculated based on actual body weight). The maximum daily dose should not exceed 400 mg for patients weighing more than 80 kg with impaired renal function [11,37–39]. To avoid prolonged buildup and increased intake, which may increase the risk of retinopathy, ototoxicity, toxic myopathy, cardiac disease, and peripheral neuropathy [40–46], due to the drug’s effect on important metabolic pathways, patients with impaired renal function and filtration rates below 30 ml/min should receive 50% of the normal hydroxychloroquine dose [47]. We have two corollaries. For patients with normal renal function, we may consider exploring the optimal dosage balance for SLE treatment, anti-infection utility, and safety. For patients with impaired renal function and concerns about dosage and renal function, a different anti-infection regimen may be more beneficial. Adherence should also be considered, as studies suggest that the benefits of adherence may outweigh the potential risks, and this medication should not be ignored [1]. The choice should be based on the patient’s medical condition.

In patients with systemic lupus erythematosus (SLE), increased disease activity is linked to further immune system dysfunction, which not only raises the risk of infections [48] but may also result in damage to vital organs. Therefore, evaluating the role of hydroxychloroquine (HCQ) in controlling SLE disease activity and preventing organ damage is crucial. Unfortunately, the studies we included did not provide sufficient data for our analysis. However, existing literature indicates that the incidence of kidney injury was significantly lower in the HCQ-treated group compared to the non-HCQ-treated group [22]. Additionally, although the SELENA-SLEDAI scores were slightly higher in the HCQ group than in the non-HCQ group, the investigators suggested that this may be due to the lower utilization of HCQ during its initial local approval period, noting that HCQ suppresses disease activity. Further literature review revealed that HCQ significantly reduced kidney damage accumulation, prevented cardiovascular disease, and effectively controlled SLE disease activity [16,49,50]. HCQ also increased the probability of sustained clinical remission (sCR), reducing the risk of SLE recurrence, which is beneficial for preventing renal impairment [51]. Recent studies have shown that with early diagnosis and standardized treatment, the leading cause of death in SLE patients has shifted from vital organ damage caused by the disease to severe infections. The proportion of deaths due to severe infections among SLE patients has steadily increased [52]. Our analysis further suggests that HCQ may significantly reduce the risk of serious infections in these patients. Therefore, HCQ plays a crucial role in reducing organ damage, improving survival, and controlling disease activity to prevent recurrence in SLE patients.

It is also worth noting that hydroxychloroquine, discovered serendipitously and not through a conventional drug development process, has a wide range of applications in rheumatic diseases, including as part of the treatment guidelines for rheumatoid arthritis (RA) and SLE [11,53]. However, based on the results of our analysis, we believe that hydroxychloroquine is more suitable for anti-infective use in SLE patients than in RA patients. This conclusion is supported by a study of three common infectious agents in RA patients—Porphyromonas gingivalis (P. gingivalis), Proteus mirabilis (P. mirabilis), and Epstein-Barr virus (EBV)—for which the inhibitory effect of hydroxychloroquine remains unclear [54]. In contrast, hydroxychloroquine has proven effective in inhibiting common pathogens in SLE patients, reducing the risk of serious infections. Therefore, while hydroxychloroquine is also used in RA treatment, its anti-infective potential appears more significant in SLE patients.

The study has some limitations. (1) The limited data, high heterogeneity of results, and potential bias present challenges. Despite including as many studies as possible and extracting data, the restricted data hindered baseline calculations, comparisons, and further analysis of hydroxychloroquine’s effects on disease activity and organ damage. Additionally, some subgroups had only one data set, so the results should be interpreted with caution.(2) The effect of smoking was not considered. Smoking (including passive smoking), a common maladaptive behavior, may inhibit the effect of HCQ [55–57]. Statistical analysis of this condition is necessary, but the included studies did not report this factor, potentially introducing bias. (3) Subgroup analysis of dose was not performed. Authoritative studies state that daily doses measured in milligrams, calculated using true body weight, are more favorable for safety, especially for long-term use. However, the included studies only used mg/day as the unit of measurement and did not provide the subjects’ weight parameters, limiting precision on the topic of dosage and safety [40]. The conclusions in this section should also be interpreted with caution.

Using eight included studies, we analyzed the effect of hydroxychloroquine on the risk of infection in SLE patients, considering infection type, analysis type, study type, and dosage. Our study concluded that hydroxychloroquine tended to reduce the risk of infection in SLE patients, although not statistically significant. Hydroxychloroquine was significantly effective in preventing serious infections and associated with a low infection risk in SLE patients. Careful consideration is required when using high doses or administering hydroxychloroquine to patients with impaired renal function. Future research should explore the optimal dose, particularly when hydroxychloroquine is used in combination with other drugs (e.g., glucocorticoids, immunosuppressants). Studies on specific diseases (e.g., herpes zoster, COVID-19) should continue, and we recommend reporting smoking and weight status of included patients to provide more rigorous evidence.

## Supporting information

S1 Table PRISMA 2020 Checklist. (PDF)

S2 Table Detailed search strategy in four databases. (DOCX)

S1 File Raw data. (XLSX)

S2 File The details of literature inclusion and exclusion. (XLSX)

S3 File The details of literature quality assessment results. (DOCX)
