# Supplementary material for: Does hydroxychloroquine reduce the risk of infection in patients with systemic lupus erythematosus? a systematic review and meta-analysis
Source: PLoS One. 2025 Mar 25;20(3):e0320353. doi: 10.1371/journal.pone.0320353 (PMC11936296; doi:10.1371/journal.pone.0320353)
Supplement: S2 Table — (DOCX) [file pone.0320353.s002.docx]

| Supplementary Table S2. Search strategy and results | | |
| --- | --- | --- |
| Database | Search strategy | Search results (number) |
| Pubmed | (((((((((Hydroxychloroquine[MeSH Terms]) OR (Hydroxychloroquine[Text Word])) OR (Oxychlorochin[Text Word])) OR (Oxychloroquine[Text Word])) OR (Hydroxychlorochin[Text Word])) OR (Plaquenil[Text Word])) OR (Hydroxychloroquine Sulfate[Text Word])) OR (Hydroxychloroquine Sulfate (1:1) Salt[Text Word])) AND (((((((Infections[MeSH Terms]) OR (Infections[Title/Abstract])) OR (Infection[Title/Abstract] AND Infestation[Title/Abstract])) OR (Infestation[Title/Abstract] AND Infection[Title/Abstract])) OR (Infections[Title/Abstract] AND Infestations[Title/Abstract])) OR (Infestations[Title/Abstract] AND Infections[Title/Abstract])) OR (Infection[Title/Abstract]))) AND ((((((((Lupus Erythematosus, Systemic[MeSH Terms]) OR (Lupus Erythematosus, Systemic[Title/Abstract])) OR (Systemic Lupus Erythematosus[Title/Abstract])) OR (Lupus Erythematosus Disseminatus[Title/Abstract])) OR (Libman-Sacks Disease[Title/Abstract])) OR (Disease, Libman-Sacks[Title/Abstract])) OR (Libman Sacks Disease[Title/Abstract])) OR (systemic lupus erythematosus[Title/Abstract])) | 340 |
| Embase | (('hydroxychloroquine'/exp OR 'hydroxychloroquine':ab,ti OR 'oxychlorochin':ab,ti OR 'oxychloroquine':ab,ti OR 'hydroxychlorochin':ab,ti OR 'plaquenil':ab,ti OR 'hydroxychloroquine sulfate':ab,ti OR 'hydroxychloroquine sulfate (1:1) salt':ab,ti) AND ('infections'/exp OR 'infections':ab,ti OR 'infection and infestation':ab,ti OR 'infestation and infection':ab,ti OR 'infections and infestations':ab,ti OR 'infestations and infections':ab,ti OR 'infection':ab,ti) AND ('lupus erythematosus, systemic'/exp OR 'lupus erythematosus, systemic':ab,ti OR 'systemic lupus erythematosus':ab,ti OR 'lupus erythematosus disseminatus':ab,ti OR 'libman-sacks disease':ab,ti OR 'disease, libman-sacks':ab,ti OR 'libman sacks disease':ab,ti OR 'systemic lupus erythematosus':ab,ti)) | 3662 |
| Web of Science | TS=(Hydroxychloroquine OR Oxychlorochin OR Oxychloroquine OR Hydroxychlorochin OR Plaquenil OR "Hydroxychloroquine Sulfate" OR "Hydroxychloroquine Sulfate (1:1) Salt") AND TS=(Infections OR "Infection and Infestation" OR "Infestation and Infection" OR "Infections and Infestations" OR "Infestations and Infections" OR Infection) AND TS=("Lupus Erythematosus, Systemic" OR "Systemic Lupus Erythematosus" OR "Lupus Erythematosus Disseminatus" OR "Libman-Sacks Disease" OR "Disease, Libman-Sacks" OR "Libman Sacks Disease" OR "systemic lupus erythematosus") | 269 |
| Cochrane | ((Hydroxychloroquine:ti,ab,kw OR Oxychlorochin:ti,ab,kw OR Oxychloroquine:ti,ab,kw OR Hydroxychlorochin:ti,ab,kw OR Plaquenil:ti,ab,kw) OR (Hydroxychloroquine Sulfate:ti,ab,kw)) AND ((Infections:ti,ab,kw OR Infection and Infestation:ti,ab,kw OR Infestation and Infection:ti,ab,kw OR Infections and Infestations:ti,ab,kw OR Infestations and Infections:ti,ab,kw) OR (Infection:ti,ab,kw)) AND ((Lupus Erythematosus, Systemic:ti,ab,kw OR Systemic Lupus Erythematosus:ti,ab,kw OR Lupus Erythematosus Disseminatus:ti,ab,kw OR Libman-Sacks Disease:ti,ab,kw OR Disease, Libman-Sacks:ti,ab,kw) OR (Libman Sacks Disease:ti,ab,kw OR systemic lupus erythematosus:ti,ab,kw)) | 20 |
