# Supplementary material for: Does hydroxychloroquine reduce the risk of infection in patients with systemic lupus erythematosus? a systematic review and meta-analysis
Source: PLoS One. 2025 Mar 25;20(3):e0320353. doi: 10.1371/journal.pone.0320353 (PMC11936296; doi:10.1371/journal.pone.0320353)
Supplement: S3 File — (DOCX) [file pone.0320353.s005.docx]

Literature Quality Assessment Results of cohort

| Study | Selection | | | | Comparability | | Outcome  Scores | | | Total Score |
| --- | --- | --- | --- | --- | --- | --- | --- | --- | --- | --- |
|  | Representiveness | Selection of non-exposed | Ascertainment of exposure | Outcome not present at start | Comparability on most important factors | Comparability on other risk factors | Assessment of outcome | Long enough follow-up(median≧1 year) | Adequacy(completenses) of follow-up |  |
| Sakai 2020 | ★ | ★ | ★ | ★ | ★ | ★ | ★ | ★ | ★ | 9 |
| Simard 2021 | ★ | ★ | ★ | ★ | ★ | ★ | ★ | ★ | ★ | 9 |
| Hidekawa 2023 |  | ★ | ★ | ★ | ★ | ★ | ★ | ★ |  | 7 |
| Sun 2023 | ★ | ★ | ★ | ★ | ★ |  | ★ |  | ★ | 7 |
| Pail2023 | ★ | ★ | ★ | ★ | ★ |  | ★ | ★ | ★ | 8 |

Literature Quality Assessment Results of Case-control

| Study | Selection | | | | Comparability | | Outcome  Scores | | | Total Score |
| --- | --- | --- | --- | --- | --- | --- | --- | --- | --- | --- |
|  | Case identification is appropriate | Case representation | Contrast selection | Determination of contrast | Important confounding factors | Other confounding factors | Identification of exposure factors | The exposure factors of cases and controls were determined by the same method | Nonresponse rate |  |
| Merayo-Chalico 2013 | ★ | ★ | ★ | ★ | ★ |  | ★ | ★ | ★ | 8 |
| Hu 2016 | ★ | ★ | ★ | ★ | ★ | ★ | ★ | ★ | ★ | 9 |
| Zamora 2020 | ★ | ★ | ★ | ★ | ★ |  | ★ | ★ | ★ | 8 |
